# Sequencing HIV Diagnostic Samples to Detect Genetic Clusters and Assess Sequence Coverage Gaps

**DOI:** 10.1093/ofid/ofaf305

**Published:** 2025-05-23

**Authors:** Cara J Broshkevitch, Shuntai Zhou, Annalea Greifinger, Kimberly Enders, Nathan Long, Erika Samoff, Kimberly A Powers, Victoria Mobley, Simon D W Frost, Erik Volz, Scott Shone, Joseph J Eron, Myron S Cohen, Ronald Swanstrom, Ann M Dennis

**Affiliations:** Department of Epidemiology, University of North Carolina at Chapel Hill, Chapel Hill, North Carolina, USA; Lineberger Comprehensive Cancer Center, University of North Carolina at Chapel Hill, Chapel Hill, North Carolina, USA; Division of Infectious Diseases, University of North Carolina at Chapel Hill, Chapel Hill, North Carolina, USA; Department of Biostatistics, University of North Carolina at Chapel Hill, Chapel Hill, North Carolina, USA; Lineberger Comprehensive Cancer Center, University of North Carolina at Chapel Hill, Chapel Hill, North Carolina, USA; Division of Public Health, North Carolina Department of Health and Human Services, Raleigh, North Carolina, USA; Department of Epidemiology, University of North Carolina at Chapel Hill, Chapel Hill, North Carolina, USA; Division of Public Health, North Carolina Department of Health and Human Services, Raleigh, North Carolina, USA; Microsoft Premonition, Microsoft Strategic Missions and Technologies, Redmond, Washington, USA; Department of Infectious Disease Epidemiology, London School of Hygiene and Tropical Medicine, London, UK; Department of Infectious Disease Epidemiology, Imperial College London, London, UK; MRC Centre for Global Infectious Disease Analysis, Imperial College London, London, UK; Division of Public Health, North Carolina Department of Health and Human Services, Raleigh, North Carolina, USA; Division of Infectious Diseases, University of North Carolina at Chapel Hill, Chapel Hill, North Carolina, USA; Institute for Global Health and Infectious Diseases, University of North Carolina at Chapel Hill, Chapel Hill, North Carolina, USA; Institute for Global Health and Infectious Diseases, University of North Carolina at Chapel Hill, Chapel Hill, North Carolina, USA; Lineberger Comprehensive Cancer Center, University of North Carolina at Chapel Hill, Chapel Hill, North Carolina, USA; Department of Biochemistry and Biophysics, University of North Carolina at Chapel Hill, Chapel Hill, North Carolina, USA; Division of Infectious Diseases, University of North Carolina at Chapel Hill, Chapel Hill, North Carolina, USA

**Keywords:** HIV, HIV diagnosis, molecular epidemiology, sequence, transmission cluster

## Abstract

**Background:**

HIV molecular cluster detection in the United States relies on HIV sequences obtained from drug resistance testing during clinical care (“routine care sequences”). This approach misses people who are not linked to care or who receive care but have uncollected or unreported sequences.

**Methods:**

We collected “HIV test sequences” from remnant serum samples of people testing newly positive from 2018 through 2021 by a large public health laboratory in North Carolina. We incorporated the HIV test sequences into a statewide molecular cluster analysis and assessed impact on “active cluster” detection (≥5 members newly diagnosed). We described data gaps filled by HIV test sequences, comparing (1) the extent of care sequence missingness due to gaps in care linkage vs sequence collection or reporting and (2) the characteristics of people with an HIV test sequence who had a care sequence, care but no care sequence, or no evidence of care.

**Results:**

Of 19 770 people included in the cluster analysis, 847 had an HIV test sequence, one-third of whom had no routine care sequence. We identified 13 additional active clusters (a 33% relative increase) and 40 larger active clusters after incorporating HIV test sequences. Most people with an HIV test sequence but no care sequence (78%) had another care indicator, suggesting sequence undercollection or underreporting, but a fifth (22%) had no evidence of care.

**Conclusions:**

Higher sequence coverage can improve cluster detection. While increased routine care sequence collection and reporting could fill many data gaps, sequencing remnant HIV test samples could include people without care linkage.

## Teaser

Incorporating sequences collected from remnant HIV test samples into molecular cluster analysis improved cluster detection. Although most newly included people received clinical care but had missing care sequence data, about one-fifth had no evidence of care linkage.

An HIV cluster may indicate rapid transmission within a sexual or drug use network inadequately reached by prevention and care services [1, 2]. If a cluster of concern is promptly identified, public health officials can prioritize HIV care for cluster members and HIV prevention for members’ sexual or injecting partners to interrupt onward HIV spread [3–6]. Molecular epidemiology identifies transmission clusters based on the similarity of HIV genetic sequences among people with diagnosed infection [7]. In the United States, providers and laboratories routinely report sequences obtained during clinical drug resistance testing to state public health departments, which transmit the deidentified sequences alongside HIV RNA viral loads and CD4+ T-lymphocyte counts to the Centers for Disease Control and Prevention (CDC) as part of the National HIV Surveillance System [8]. Molecular cluster analysis has characterized US HIV transmission networks at national [2, 9] and local [10–15] levels and detailed the scope and timing of cluster growth during recent outbreaks [16–18].

Different molecular cluster definitions may support cluster response vs monitoring. For example, the CDC aims to detect and respond to “priority clusters,” defined as clusters with ≥3 to 5 members newly diagnosed in the prior year, using a close genetic distance threshold ≤0.5%, indicating recent and rapid transmission [19]. By comparison, monitoring efforts may track cluster growth over a longer time frame and use a looser genetic distance threshold (ie, 1.5%) to more broadly include epidemiologically related HIV transmissions [20].

Molecular cluster detection strategies fundamentally rely on sequence completeness. Genotypic drug resistance testing is widely recommended at HIV care entry to direct antiretroviral therapy selection [21], producing large volumes of sequence data that can be analyzed for public health purposes. However, cluster detection strategies based on sequences collected during clinical care have missing or delayed data (1) when people with HIV do not receive drug resistance testing (ie, they are undiagnosed or not linked to care, or the provider does not order the test) or (2) if sequences are not reported to surveillance systems [10]. Moderate sequence completeness (about 50% of people diagnosed with HIV) is sufficient to support cluster detection, but more complete data can provide a more nuanced understanding of a local epidemic [2, 22]. In simulation studies with US surveillance data, subsampling existing sequence data lowered sensitivity to detect CDC-defined priority clusters [23, 24]. More work is needed to quantify the potential impact of incorporating missing sequences on real-world cluster detection.

We increased sequence completeness by collecting sequences earlier in the HIV care cascade, leveraging sequences generated by the PROMPT research study (Phylodynamics for Response and Monitoring of HIV Transmission) from remnant HIV test samples (“HIV test sequences”) of people newly diagnosed by a large public health laboratory. We hypothesized that incorporating HIV test sequences would enhance cluster detection by including people who were not linked to care, whose care provider did not order drug resistance testing, or whose “routine care sequence” from drug resistance testing was unreported. To examine this hypothesis, we conducted a statewide molecular cluster analysis, quantifying the impact of incorporating HIV test sequences on detection of active clusters for monitoring, defined as clusters with ≥5 members newly diagnosed between 2018 and 2021 (prior 4 years) using a genetic distance threshold <1.5%. To understand data gaps filled by incorporating the HIV test sequences, we (1) assessed the extent to which sequence undercollection or underreporting vs lack of care linkage drove routine care sequence missingness among people with an HIV test sequence and (2) compared demographic characteristics and indicators of health system engagement across people with an HIV test sequence who had a reported care sequence, were linked to care without a reported care sequence, or had no evidence of care in the study time frame.

## METHODS

### Public Health and Routine Care Sequence Reporting Practices in North Carolina

People newly diagnosed with HIV in North Carolina (NC) are referred to the NC Division of Public Health (DPH) for initial interview, partner services, and care linkage assistance (bridge counseling). Demographic data, HIV viral loads, and CD4+ T-cell counts, and other data from laboratory, clinician, or interview reports are recorded in the NC Electronic Disease Surveillance System (NC EDSS). These data include care sequences that are routinely reported by commercial laboratories statewide. Although care sequence reporting was mandated in 2018, some sequences deposited in NC EDSS were collected as early as 2010 [25].

### Parent Study Design

PROMPT was a National Institutes of Health–funded study designed to detect and monitor HIV genetic clusters in NC [11, 26]. From 2018 through 2023, demographic, clinical, and public health surveillance data for people newly diagnosed with HIV, aged ≥13 years, and with ≥1 HIV *pol* sequence were extracted monthly from the NC EDSS and incorporated into the PROMPT dataset. Multiplexed Primer-ID next-generation sequencing (NGS) was performed on remnant serum specimens (“HIV test sequences”) of all people testing newly positive at the NC State Laboratory of Public Health (NC-SLPH) between 2018 and 2021, as previously described [26, 27]. The NC-SLPH processes HIV screening tests distributed by the NC DPH or an associated testing and counseling site, representing about 25% of people newly diagnosed in NC [28]. As HIV test sequences were collected through PROMPT in collaboration with the NC DPH and not through clinical care, HIV test sequences were not reported to clinicians. NGS was performed on specimens collected within 30 days of the diagnosis date recorded by the NC DPH at the time of sequencing to ensure that people were newly diagnosed [26, 27]. For data privacy reasons, only consensus-level sequences from NGS (comparable to routine care sequences) were used for cluster analysis [11, 26].

### Evaluating the Impact of HIV Test Sequences on Active Cluster Detection

**Figure 1.**
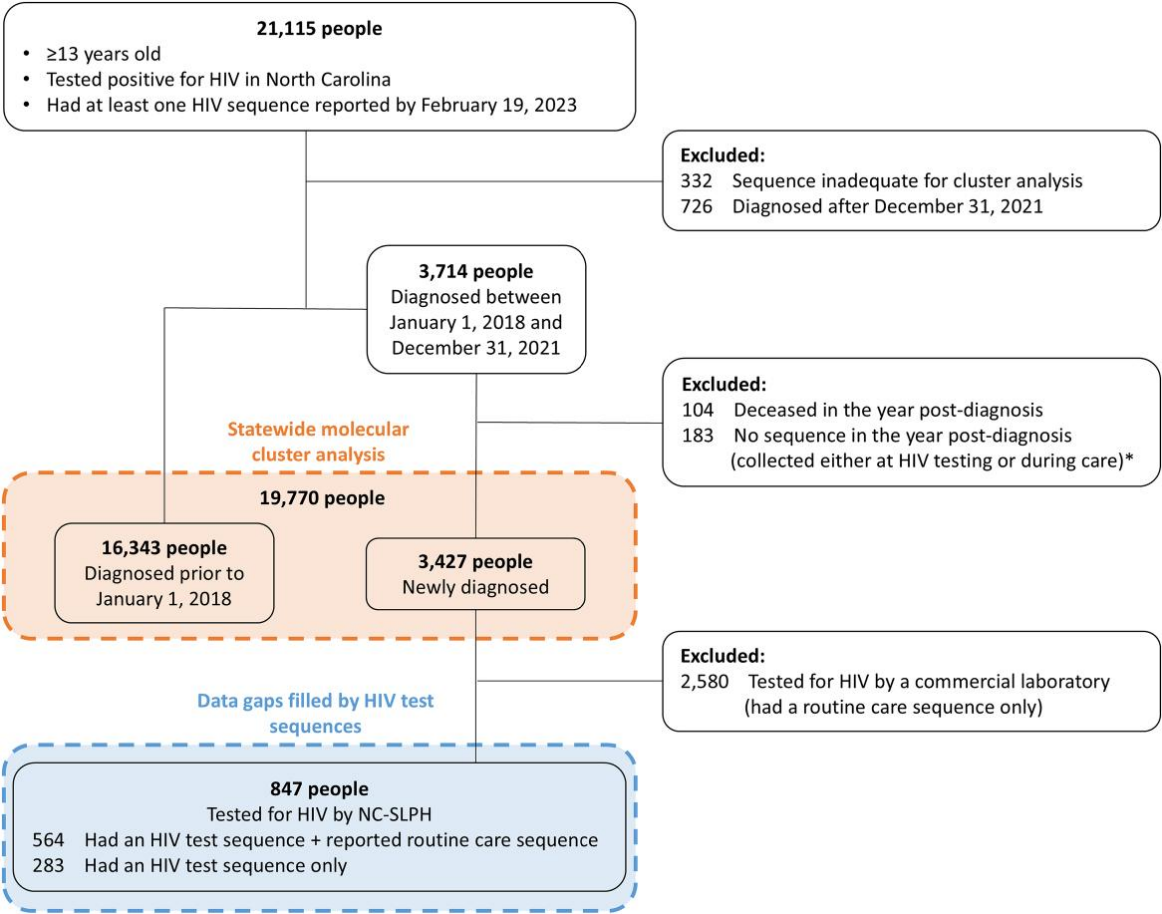
Analysis flow diagram. The source population for this analysis was people aged ≥13 years and diagnosed with HIV in North Carolina, with at least 1 sequence (HIV test and/or routine care sequence) reported to public health by 19 February 2023. The statewide molecular cluster analysis included 19 770 people (1) who were diagnosed prior to 1 January 2018 and had an adequate sequence or (2) who were newly diagnosed with HIV between 2018 and 2021 and were alive and had an adequate sequence within 1 year of HIV diagnosis. The analysis of data gaps filled by HIV test sequences included 847 people who were newly diagnosed with HIV by the North Carolina State Laboratory of Public Health (NC-SLPH) between 2018 and 2021, had an HIV test sequence adequate for cluster analysis, and were alive within 1 year of HIV diagnosis. *Modified in sensitivity analysis (Supplementary Methods).

We evaluated PROMPT data for people aged ≥13 years who tested positive for HIV in NC and had an available sequence (HIV test and/or routine care sequence, N = 21 115 people; Figure 1). We analyzed data in the NC EDSS by 19 February 2023 to allow 1 year of follow-up after diagnosis plus ≥1 month for reporting delays. We excluded 332 people with an inadequate sequence for cluster analysis (lacked RT region of the *pol* gene), as well as 726 people diagnosed after 31 December 2021. Among people diagnosed between 2018 and 2021, we made additional exclusions to ensure consistent follow-up time for care sequencing or care linkage across diagnosis years in our analysis of data gaps filled by HIV test sequences. Specifically, we excluded 104 people deceased in the year postdiagnosis: as we accessed only the death year, we excluded those deceased in the same or subsequent calendar year as their diagnosis date. We also included only sequences collected within 1 year postdiagnosis, excluding 183 people without a sequence in the year postdiagnosis. In a sensitivity analysis, we relaxed this 1-year restriction on follow-up time for care sequencing and care linkage (Supplementary Methods). In total, the cluster analysis included 19 770 people: 16 343 were diagnosed prior to 1 January 2018 and had an adequate sequence, and 3427 were newly diagnosed in the 2018–2021 period and were alive and had an adequate sequence 1 year postdiagnosis.

We used an automated molecular cluster analysis system (nextHIV2) to construct transmission networks [11]. We aligned sequences covering a partial *pol* region (821 nucleotides) and used the TN-93 nucleotide substitution model [29] with averaging of ambiguities to estimate pairwise genetic distances. We defined pairs as those with <1.5% distance, selecting the shortest distance for people with multiple sequences. Clusters included at least 1 linked pair.

To assess the impact on cluster detection of augmenting routine care sequences with HIV test sequences, we identified clusters that included at least 1 person without a care sequence reported in the year postdiagnosis who was newly linked to the cluster by an HIV test sequence. We quantified impact on detection of active clusters for monitoring, defined as clusters with ≥5 members newly diagnosed between 2018 and 2021, using a genetic distance threshold <1.5%. In a supplemental analysis, we evaluated the impact on detection of CDC priority clusters, defined as clusters with ≥5 members newly diagnosed in the prior year, using a threshold ≤0.5%.

### Describing Data Gaps Filled by HIV Test Sequences

To understand data gaps filled by incorporating additional sequences into the molecular cluster analysis, we conducted a series of analyses among people with an HIV test sequence. First, we assessed the extent to which sequence undercollection and underreporting vs lack of care linkage drove routine care sequence missingness. We classified a person's routine care sequence as uncollected or unreported if that person had another care linkage indicator (≥1 reported HIV viral load or CD4+ T-cell count) within the year postdiagnosis. We grouped sequence undercollection and underreporting because these processes cannot be distinguished by surveillance data. People with no care indicators in the year postdiagnosis were classified as not linked to care.

We then compared distributions of demographic characteristics and indicators of health system engagement across people with an HIV test sequence who had a reported routine care sequence, care linkage but no routine care sequence, and no care linkage or routine care sequence. Specifically, we compared demographic and geographic characteristics associated with known or expected disparities in NC HIV burden [30]. Indicators of health care and public health system engagement included proportions of people interviewed by DPH staff for partner services and/or assigned bridge counseling at diagnosis for care linkage. Additionally, among those with a viral load reported within 1 year postdiagnosis, we compared the proportion virally suppressed (defined as ≤200 copies of HIV RNA per milliliter of blood). Finally, we compared distributions in recency of HIV infection at the time of HIV test sequence collection, a metric enabled by the multiplexed Primer ID–NGS technology used to generate HIV test sequences. We hypothesized that people missing routine care sequences due to undercollection or underreporting would have similar demographic characteristics and health system engagement as those with a reported routine care sequence, while people missing a routine care sequence due to lack of care linkage would differ in ways potentially indicating more limited public health and clinical engagement. We calculated 2-sided *P* values using a *t* test for continuous variables and χ^2^ test for categorical variables with an α value of 0.05. Statistical analyses were performed in R version 4.1.1.

## RESULTS

Of 19 770 people in the molecular cluster analysis, 847 tested newly positive for HIV by the NC-SLPH from 2018 through 2021 and had an HIV test sequence collected from their remnant HIV test sample (Figure 1). One-third (n = 283) of these 847 people did not have a routine care sequence reported within the year postdiagnosis. Of those with an HIV test and routine care sequence, the median time from HIV test to routine care sequence collection was 27 days (IQR, 18–47; Supplementary Figure 1).

### Impact of HIV Test Sequences on Active Cluster Detection

Overall, there were 704 unique clusters identified statewide, with a median total size of 3 members (range, 2–108) and a median 2 members diagnosed between 2018 and 2021 (range, 1–44). Of these 704 clusters, 313 (44%) included ≥1 member with an HIV test sequence, 134 (43%) of which included additional members identified by an HIV test sequence alone (Supplementary Figure 2).

**Figure 2.**
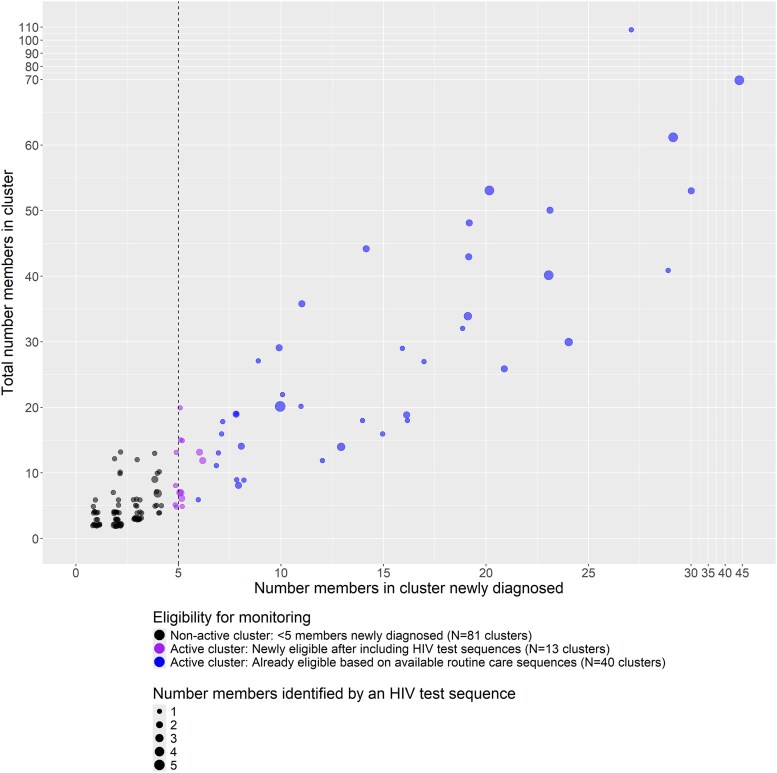
Eligibility for monitoring of 134 clusters that included ≥1 member newly diagnosed with HIV by the North Carolina State Laboratory of Public Health with an HIV test sequence but no reported routine care sequence. Active clusters had ≥5 members (dotted line) newly diagnosed between 2018 and 2021, with clusters defined by a genetic distance threshold <1.5%.

Under a public health approach in which active clusters with ≥5 members newly diagnosed between 2018 and 2021 would have been monitored, 53 of the 134 clusters would have been eligible for monitoring (Figure 2). Of these 53 active clusters, 40 already met criteria with available routine care sequences but were larger after incorporating HIV test sequences. These 40 clusters had a median total size of 24 members (range, 6–108), with a median 14 members newly diagnosed (range, 6–44; Supplementary Table 1). The 13 additional active clusters that were newly eligible for monitoring after including HIV test sequences represented a 33% relative increase in identified active clusters. These 13 additional clusters were of moderate total size (median [range], 8 [5–20] members and 5 [5–6] newly diagnosed) and included 1 or 2 members identified by an HIV test sequence. In the sensitivity analysis relaxing the 1-year restriction on follow-up time for care sequencing, there was a similar percentage relative increase in identified active clusters (28%; Supplementary Results). Although we identified 4 CDC-defined priority clusters in NC, none included additional members identified by an HIV test sequence alone.

### Data Gaps Filled by HIV Test Sequences

Most of the 283 people with an HIV test sequence but no reported routine care sequence (78%, n = 220) had another HIV care indicator within the year postdiagnosis (Table 1), suggesting sequence undercollection or underreporting. However, one-fifth of people with an HIV test sequence but no routine care sequence (22%, n = 63) had no care indicators and thus were unlikely to have linked to care in NC in the year postdiagnosis. In the sensitivity analysis relaxing the 1-year restriction on follow-up time for care sequencing and care linkage, most people without a routine care sequence again had care indicators (87%; Supplementary Results).

**Table 1.** Demographic, Geographic, and Cluster Characteristics Among People With an HIV Test Sequence, Stratified by Presence of a Reported Routine Care Sequence or Indicators of HIV Care Linkage

	Reported Routine Care Sequence (n = 564/847, 67%)	No Reported Routine Care Sequence (n = 283/847, 33%)		
	Care Indicator (n = 564/564, 100%)	Care Indicator (n = 220/283, 78%)	No Care Indicator (n = 63/283, 22%)	All (n = 283/283, 100%)
Identified in a cluster				
Yes	412 (73)^a^	140 (64)	41 (65)	181 (64)
No	152 (27)	80 (36)	22 (35)	102 (36)
Identified in an active cluster^b^				
Yes	222 (54)	76 (54)	16 (39)	92 (51)
No	190 (46)	64 (46)	25 (61)	89 (49)
Missing (not in a cluster)	152	80	22	102
Gender^c^				
Man	485 (86)	181 (82)	51 (81)	232 (82)
Women	67 (12)	34 (16)	9 (14)	43 (15)
Transgender man	1 (1)	0 (0)	0 (0)	0 (0)
Transgender woman	11 (2)	5 (2)	3 (5)	8 (3)
Age at diagnosis, y				
Median (range)	26 (16–81)	28 (16–69)	29 (19–58)	28 (16–69)
Race and ethnicity^d^				
Black, non-Hispanic	380 (67)	132 (60)	42 (67)	174 (62)
White, non-Hispanic	86 (15)	42 (19)	10 (16)	52 (18)
Hispanic	61 (11)	27 (12)	5 (8)	32 (11)
Other	10 (2)	5 (2)	2 (3)	7 (3)
Missing	27 (5)	14 (6)	4 (6)	18 (6)
HIV risk group^e^				
MSM	405 (72)	147 (67)	36 (57)	183 (65)
PWID	11 (2)	6 (3)	1 (2)	7 (3)
MSM-PWID	17 (3)	5 (2)	0 (0)	5 (3)
Heterosexual	112 (20)	48 (22)	21 (33)	69 (24)
Missing	19 (3)	14 (6)	5 (8)	19 (7)
HIV diagnosis year				
2018	186 (33)	76 (35)	22 (35)	98 (35)
2019	147 (26)	81 (37)	15 (24)	96 (34)
2020	90 (16)	23 (11)	11 (18)	34 (12)
2021	141 (25)	40 (18)	15 (24)	55 (19)
Diagnosed in North Carolina^f^				
Yes	556 (99)	208 (95)	57 (91)	265 (94)
No	8 (1)	12 (6)	6 (10)	18 (6)
Region of residence at diagnosis^g^				
Asheville	32 (6)	26 (12)	3 (5)	29 (10)
Charlotte	48 (9)	25 (11)	10 (16)	35 (12)
Fayetteville	64 (11)	29 (13)	6 (10)	35 (12)
Greensboro	130 (23)	69 (31)	25 (40)	94 (33)
Raleigh	148 (26)	36 (16)	7 (11)	43 (15)
Wilmington	34 (6)	7 (3)	5 (8)	12 (4)
Winterville	108 (19)	28 (13)	7 (11)	35 (12)
Rurality of county at diagnosis^h^				
Rural	87 (15)	67 (31)	17 (27)	84 (30)
Urban	477 (85)	153 (70)	46 (73)	199 (70)

HIV test sequences were obtained from remnant serum samples of people testing newly positive for HIV by the NC-SLPH between 2018 and 2021. Follow-up time was within 1 year of HIV diagnosis for collection of routine care sequence and indicators of care linkage (defined as a reported HIV viral load or CD4+ T-cell count).

Abbreviations: DPH, Division of Public Health; MSM, men who have sex with men; NC, North Carolina; NC-SLPH, North Carolina State Laboratory of Public Health; PWID, person who injects drugs.

^a^Data are presented as No. (%) unless noted otherwise. Percentages are based on care indicator numerator and may not add to 100 due to rounding.

^b^Active clusters were defined as clusters with ≥5 members newly diagnosed between 2018 and 2021, with clusters defined by a genetic distance threshold <1.5%.

^c^Gender was reported as categorical data to the NC DPH.

^d^Race and ethnicity were reported as categorical data to the NC DPH, combined into a single variable, and included in the analysis to assess health disparities. We collapsed American Indian/Alaska Native and Asian/Pacific Islander into the “other” category due to small cell counts and to match levels used in previous analyses. This group is likely highly heterogeneous.

^e^HIV risk group was derived by assigning people their most likely transmission category based on exposure data shared during interviews by NC DPH staff at or near the time of diagnosis.

^f^State of diagnosis could be reported as outside NC if a person was retested for HIV at NC-SLPH shortly after relocating to NC or was tested at NC-SLPH but was discovered to be residing outside NC.

^g^Region of residence was based on a person's county at diagnosis or upon moving to NC.

^h^The 2013 National Center for Health Statistics Urban-Rural Classification Scheme for Counties was used to dichotomize county of residence at HIV diagnosis as rural (noncore, micropolitan) or urban (small metro, medium metro, large fringe metro, large central metro) [31].

People without a reported routine care sequence but with another care indicator generally resembled people with a routine care sequence. Both groups were mostly cis-gender men, Black and non-Hispanic, and reported sex with men (Table 1). However, distributions of diagnosis years differed (*P* = .005), with a higher percentage (71%, n = 157) of the 220 people with no routine care sequence but with care indicators diagnosed in the 2 earlier years (2018–2019) than those with a routine care sequence (59%, 333/564). People with no routine care sequence but with care indicators were also less likely than those with a care sequence to have their state of diagnosis reported as NC (95% vs 99%, *P* = .003). This could occur if a person was retested for HIV at the NC-SLPH shortly after relocating to NC or was tested at NC-SLPH but found to be residing outside NC. At the time of diagnosis, people with no routine care sequence but with care indicators resided in different regions than those with a routine care sequence (7-category *P* < .001) and were more likely to reside in a rural county (*P* < .001). Both groups had similar levels of public health and clinical engagement, although fewer people without a routine care sequence were interviewed by DPH for partner services (90% vs 95%, *P* = .01; Table 2).

**Table 2.** Indicators of Public Health and Clinical Engagement Among People With an HIV Test Sequence, Stratified by the Presence of a Reported Routine Care Sequence or Indicators of HIV Care Linkage

	Reported Routine Care Sequence (n = 564/847, 67%)	No Reported Routine Care Sequence (n = 283/847, 33%)		
	Care Indicator (n = 564/564, 100%)	Care Indicator (n = 220/283, 78%)	No Care Indicator (n = 63/283, 22%)	All (n = 283/283, 100%)
Most recent viral load				
Suppressed	481 (85)^a^	175 (80)	0 (0)	175 (62)
Unsuppressed	83 (15)	43 (20)	0 (0)	43 (15)
Missing (no reported viral load)	0 (0)	2 (1)	63 (100)	65 (23)
Recency of infection^b^				
Recent	219 (39)	90 (41)	17 (27)	107 (38)
Chronic	277 (49)	98 (45)	34 (54)	132 (47)
Indeterminant	68 (12)	32 (15)	12 (19)	44 (16)
Interviewed by DPH staff for partner services				
Yes	536 (95)	198 (90)	43 (68)	241 (85)
No	20 (4)	12 (6)	15 (24)	27 (10)
Missing	3 (1)	1 (1)	1 (2)	1 (1)
Should not have had DPH interview	5 (1)	9 (4)	4 (6)	13 (5)
Ever assigned bridge counseling at diagnosis for linkage to care^c^				
Yes	512 (91)	197 (90)	49 (78)	246 (87)
No	52 (9)	23 (11)	14 (22)	37 (13)
Bridge counseling for linkage outcome^c^				
Already in care	382 (75)	138 (70)	2 (4)	140 (57)
Initiated/renewed in care	93 (18)	35 (18)	1 (2)	36 (15)
Refusal	10 (2)	1 (0.5)	20 (41)	21 (9)
Unable to locate	5 (1)	4 (2)	18 (37)	22 (9)
Out of state	4 (1)	11 (6)	7 (14)	18 (7)
Incarcerated	1 (1)	4 (2)	0 (0)	4 (2)
Other	17 (3)	4 (2)	1 (2)	5 (2)
Missing (never assigned)	52	23	14	37

HIV test sequences were obtained from remnant serum samples of people testing newly positive for HIV by the North Carolina State Laboratory of Public Health between 2018 and 2021. Follow-up time was within 1 year of HIV diagnosis for collection of routine care sequence, indicators of care linkage (defined as a reported HIV viral load or CD4+ T-cell count), most recent HIV viral load, and bridge counselor assignment.

Abbreviation: DPH, Division of Public Health.

^a^Data are presented as No. (%). Percentages are based on care indicator numerator and may not add to 100 due to rounding.

^b^ *Recent*, if remnant diagnostic specimen used for next-generation sequencing was estimated to have been collected within 9 months of HIV infection; *chronic*, if diagnostic specimen collection date was >9 months after HIV infection.

^c^Disqualifying characteristics for bridge counseling include being already in care, deceased, incarcerated, or not currently living in North Carolina. Included bridge counselor assignments within 1 year of HIV diagnosis.

People without a routine care sequence or care indicators were more likely to report heterosexual exposure and less likely to be men who have sex with men as compared with those with a routine care sequence (5-category *P* = .03; Table 1). Geographic characteristics of people with no routine care sequence or care indicators differed from those of people with a routine care sequence in similar ways as seen among people with care indicators but no routine care sequence. People without a routine care sequence or care indicators exhibited several markers of decreased public health and clinical engagement relative to those with care indicators (Table 2; Supplementary Table 2). This group tended to have less recent infections (27% diagnosed ≤9 months from HIV infection) as compared with those with care indicators, without (41%) or with (39%) a routine care sequence (*P* = .1 for both comparisons). Many were never interviewed by the DPH (24%). Of those without care indicators but assigned bridge counseling for linkage (78%), most refused care (41%), were unable to be located (37%), or were out of state (14%).

## DISCUSSION

We evaluated impact on cluster detection and described data gaps filled by incorporating sequences collected at the time of HIV testing from people diagnosed by a large public health laboratory into our molecular cluster analysis. One-third of people with an HIV test sequence did not have a reported routine care sequence obtained from drug resistance testing and were therefore newly represented in the cluster analysis. We identified 13 active clusters newly eligible for monitoring (a 33% relative increase in identified active clusters), as well as 40 larger active clusters. The 13 additional active clusters were of moderate size (median, 8 total members and 5 newly diagnosed) as compared with the 40 existing active clusters (median, 24 total members and 14 newly diagnosed). Most people newly incorporated into the cluster analysis likely had an uncollected or unreported routine care sequence at care entry, but a fifth were likely never linked to care in the year postdiagnosis. By including HIV test sequences for people without a routine care sequence, we integrated into our cluster analysis people diagnosed in earlier years and more rural NC counties, as well as those less engaged with health systems or more transiently residing in NC.

We found that incorporating HIV test sequences into the statewide cluster analysis identified additional active clusters, providing real-world evidence to support prior simulations indicating that increased sequence completeness improved CDC-defined priority cluster detection [23, 24]. While these simulations subsampled existing care sequence data [23, 24], our analysis added sequences from people linked to care without a reported care sequence and those without care indicators. People without care indicators were frequently identified in a cluster but, perhaps due to a higher proportion of chronic infections, less often linked to an active cluster than those with care indicators: people with chronic infection cluster less than people with recent infection [27, 32]. A limitation of our analysis is that we did not sequence diagnostic specimens from commercial laboratories and therefore could not estimate the impact on active cluster detection of incorporating HIV test sequences from all people newly diagnosed in NC.

We defined active clusters as those with ≥5 members newly diagnosed between 2018 and 2021, using a genetic distance threshold <1.5%. Prior studies simulating the impact of sequence completeness on cluster detection applied a narrower definition of ≥2 to 5 members newly diagnosed in the prior year, using a threshold ≤0.5% [23, 24], reflecting the CDC outbreak definition. However, a recent analysis demonstrated that 3 in 4 NC clusters, including people diagnosed between 2018 and 2021 and using a threshold ≤1.5%, had ≥1 person with recent infection (ie, diagnosed ≤9 months from transmission) [27], supporting that a 4-year time frame and 1.5% genetic distance threshold generate clusters relevant for monitoring. Jurisdictions should define a detection, monitoring, and response strategy based on their capacity to intervene on identified clusters [10].

Our observed 67% routine care sequence coverage among people diagnosed with HIV by the NC-SLPH aligns with prior reports, as does our interpretation that undercollection and underreporting drove missingness of routine care sequences. Sequence coverage was 63% among people diagnosed in NC in 2014 to 2018 [11] and ranged from 45% to 63% in other states with mandatory reporting over a similar period [1, 10, 13–15]. A study of people diagnosed in Maryland, albeit with lower sequence coverage (29%), similarly found that a lack of care linkage explained a subset (14%) of missing sequences but not all [33]. A limitation of our analysis is that we cannot distinguish between care sequence undercollection and underreporting in surveillance data. Analyses of electronic health records could further explain these data gaps.

Incorporating people with an HIV test sequence into our cluster analysis revealed temporal and geographic gaps in routine care sequence reporting, as well as HIV care gaps. People with an HIV test sequence and routine care sequence were diagnosed in later years than those with care indicators but no care sequence, suggesting that care sequence reporting improved with time. Geographic region and rurality of residence at diagnosis also differed by receipt of care sequencing, indicating potential differences in care practices or varied reporting across commercial laboratories. Other studies similarly noted varied sequence completeness across regions of Maryland [33] and higher completeness in more populated areas [34]. Uniquely, we incorporated sequence data from people without evidence of HIV care.

Our estimates of impact on cluster detection and data gaps filled by HIV test sequences best generalize to populations with similar HIV transmission dynamics and levels of diagnosis, care, and sequencing. People with diagnoses reported through the NC-SLPH attended public testing venues or were reached through partner services, representing about 25% of people newly diagnosed in NC (Supplementary Table 3 compares demographic characteristics by HIV test–processing location). Although the NC-SLPH processed diagnostic tests from all state regions, Mecklenburg County, which is a federal End the HIV Epidemic initiative county, increased testing through commercial laboratories during the study period and was underrepresented in our analysis.

While our results emphasize that increased routine care sequence collection and reporting are the first step to improving molecular cluster detection, sequencing at the time of HIV testing offers additional benefits. Sequencing remnant HIV test samples by multiplexed Primer ID–NGS, as done in this study, measures recency of HIV infection [26]. Additionally, while guidelines support drug resistance testing at HIV care entry [21] and there is evidence of continued clinical need [35], there has been recent debate about clinical benefit and cost-effectiveness with the shift toward integrase strand transfer inhibitors for first-line antiretroviral therapy [36]. If drug resistance testing declines in response to these concerns, sequencing remnant HIV test specimens could allow surveillance for transmitted drug resistance mutations, a public health activity very likely to continue [37–39]. Prior to sequencing remnant diagnostic specimens for molecular cluster investigation, programs should carefully consider whether explicit consent is needed [40].

In conclusion, sequencing remnant HIV test samples can increase sequence coverage and improve detection of active clusters, particularly moderately sized active clusters. Increased collection and laboratory reporting of routine care sequences obtained from drug resistance testing can fill most sequence data gaps. Sequencing remnant HIV test samples can additionally include people without care linkage.

## Supplementary Material

ofaf305_Supplementary_Data

## Supplementary Data

Supplementary materials are available at *Open Forum Infectious Diseases* online. Consisting of data provided by the authors to benefit the reader, the posted materials are not copyedited and are the sole responsibility of the authors, so questions or comments should be addressed to the corresponding author.

## Notes

***Acknowledgments.*** We thank the staff of the North Carolina Communicable Disease Branch and the staff of the North Carolina Laboratory of Public Health.

***Author contributions.*** C. J. B. and A. M. D. had full access to all the data in the study and take responsibility for the integrity of the data and accuracy of the data analysis. *Concept and design:* C. J. B. and A. M. D. conceptualized the analysis. *Acquisition, analysis, or interpretation of data:* N. L. and S. Z. performed the next-generation sequencing. C. J. B. performed the formal analysis. All authors contributed to interpretation of the data. *Drafting the manuscript:* C. J. B. wrote the first draft of the manuscript. A. M. D. provided drafting assistance. *Critical review of the manuscript for important intellectual content:* All authors critically reviewed the manuscript. *Statistical analysis:* C. J. B. performed the statistical analysis. *Obtained funding:* A. M. D., R. S., M. S. C., J. J. E., K. A. P., and S. Z. designed PROMPT and obtained funding. A. M. D. was the principal investigator for the PROMPT study. *Administrative, technical, or material support:* A. G. and K. E. provided data curation and cleaning. *Supervision:* A. M. D. was the principal investigator for the present analysis.

***Data-sharing statement.*** Data are available pursuant to a data access agreement with the North Carolina Division of Public Health. Data dictionary and statistical analysis code are available by request.

***Meeting presentation.*** Presented in part at the Conference on Retroviruses and Opportunistic Infections; Seattle, Washington; February 2023. Abstract 1349596, poster 855: “HIV Sequencing at Diagnosis Enhances Detection of Clusters and Potential Care Gaps.”

***Patient consent statement.*** This study does not include factors necessitating patient consent.

***Role of the funder.*** The funder had no role in the design and conduct of the study; collection, management, analysis, and interpretation of the data; preparation, review, or approval of the manuscript; and decision to submit the manuscript for publication.

***Financial support.*** This work was supported by the National Institutes of Health (R01 AI135970 and R01 AI169602 to A. M. D., R01 AI140970 and P30AI050410 to R. S., 3P30AI050410-26S1 [University of North Carolina Center for AIDS Research Ending the HIV Epidemic Administrative Supplement] to S. Z., and T32 AI007001 to C. J. B.).
