# Supplementary material for: Sequencing HIV Diagnostic Samples to Detect Genetic Clusters and Assess Sequence Coverage Gaps
Source: Open Forum Infect Dis. 2025 May 23;12(6):ofaf305. doi: 10.1093/ofid/ofaf305 (PMC12147020; doi:10.1093/ofid/ofaf305)
Supplement: ofaf305_Supplementary_Data [file ofaf305_supplementary_data.docx]

**Supplemental Appendix to:**

**Sequencing HIV diagnostic samples to detect genetic clusters and assess sequence coverage gaps**

**Authors:** Cara J. Broshkevitch, Shuntai Zhou, Annalea Greifinger, Kimberly Enders, Nathan Long, Erika Samoff, Kimberly Powers, Victoria Mobley, Simon D.W. Frost, Erik Volz, Scott Shone, Joseph J. Eron, Myron S. Cohen, Ronald Swanstrom, Ann M. Dennis

**SUPPLEMENTAL METHODS**

**Sensitivity analysis- one-year restriction on follow-up time.**

In a sensitivity analysis, we relaxed the one-year restriction on follow-up time for routine care sequences and clinical outcomes to understand how this window impacted results. In the sensitivity analysis, we excluded people deceased prior to the end of data collection (February 19, 2023). We continued to exclude HIV test sequences with a collection date more than a year from the HIV diagnosis date recorded by February 19, 2023, as these people were likely not truly newly diagnosed. (NGS was performed on residual diagnostic specimens with a collection date within 30 days of the recorded diagnosis date at the time of sequencing; however, the diagnosis date could be corrected over time.) Of the 183 people excluded in the primary analysis for no sequence within one year of HIV diagnosis:

- 174 people diagnosed with HIV by a commercial lab had a routine care sequence and were still alive by February 19, 2023, and
- 2 people diagnosed with HIV by the NC-SLPH with NGS >1 year from HIV diagnosis had a routine care sequence and were still alive by February 19, 2023.

Additionally, 29 people diagnosed with HIV by the NC-SLPH and without a routine care sequence in the primary analysis were alive and had a routine care sequence by February 19, 2023.

Overall, of the 3,714 people with an adequate sequence who were diagnosed with HIV between January 1, 2018 and December 31, 2021, only 140 (3.8%) died prior to February 19, 2023; we therefore did not expect exclusion of these people to create substantial bias. Of the remaining 3,574 people, 2,736 were tested for HIV by a commercial laboratory, leaving 838 people newly diagnosed with HIV by the NC-SLPH.

**SUPPLEMENTAL RESULTS**

**Sensitivity analysis- one-year restriction on follow-up time.**

Of the 838 people newly diagnosed with HIV by NC-SLPH: 588 people (70%) had a routine care sequence reported by February 19, 2023, and 250 people (30%) had no reported routine care sequence. One person with a reported routine care sequence had no additional care indicators (0.2%) and 33 people had no reported routine care sequence or care indicators (13%), most of whom (14 people, 42%) were diagnosed in 2021. There were 50 active clusters eligible for monitoring of 118 clusters with a member identified by an HIV test sequence. Of these 50 clusters, 39 met criteria based on available routine care sequences. We identified an additional 11 active clusters for monitoring by including HIV test sequences (a 28% increase in active clusters).

**Sensitivity analysis- CDC priority cluster definition.**

When we narrowed the cluster genetic distance threshold to ≤0.5%, as used by the CDC, we identified 129 unique clusters (Median: 2 [Range: 2-34] total members; 1 [1-7] newly diagnosed), four of which included ≥5 members newly diagnosed with HIV in the prior year (“CDC priority clusters”; 19 [8-29] total members; 6 [5-7] newly diagnosed). Three of these priority clusters included ≥1 member newly diagnosed with HIV by the NC-SLPH with an HIV test sequence (21 [16-29] total members; 6 [5-7] newly diagnosed), but none included additional members identified by an HIV test sequence alone.

**
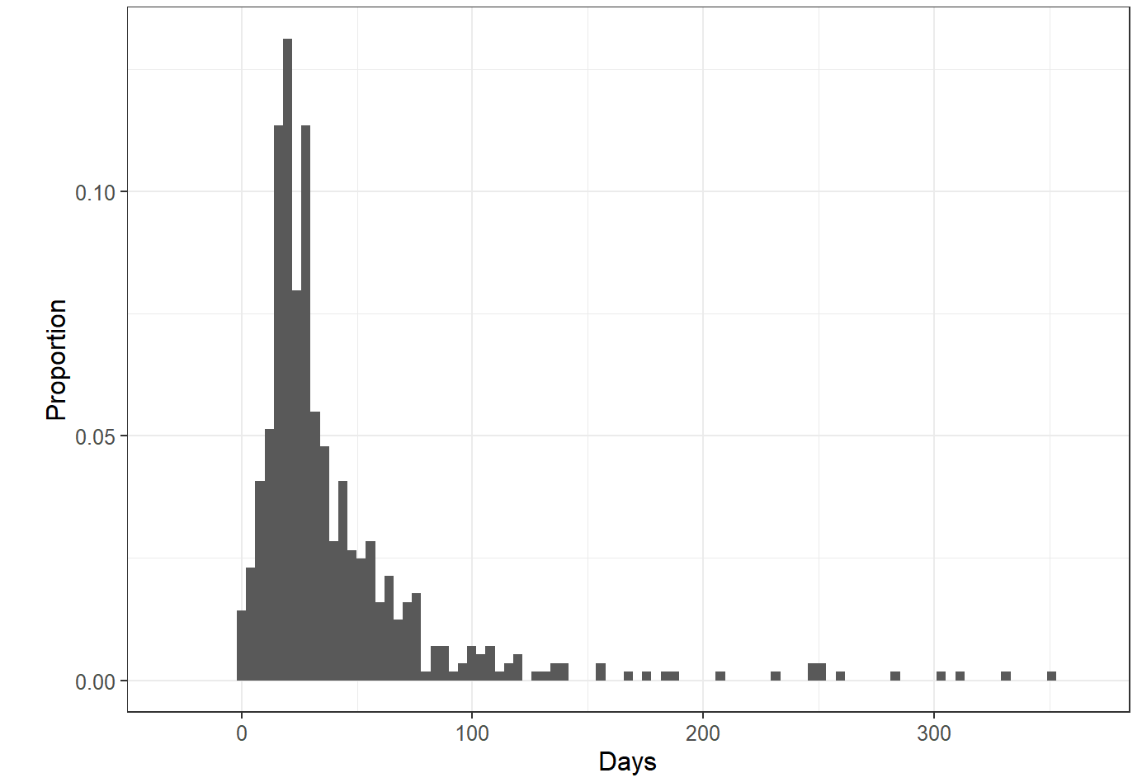
**

**Supplemental Figure 1. Time from date of HIV diagnosis to date of first routine care sequence sample collection, among people with an HIV test sequence.**

**
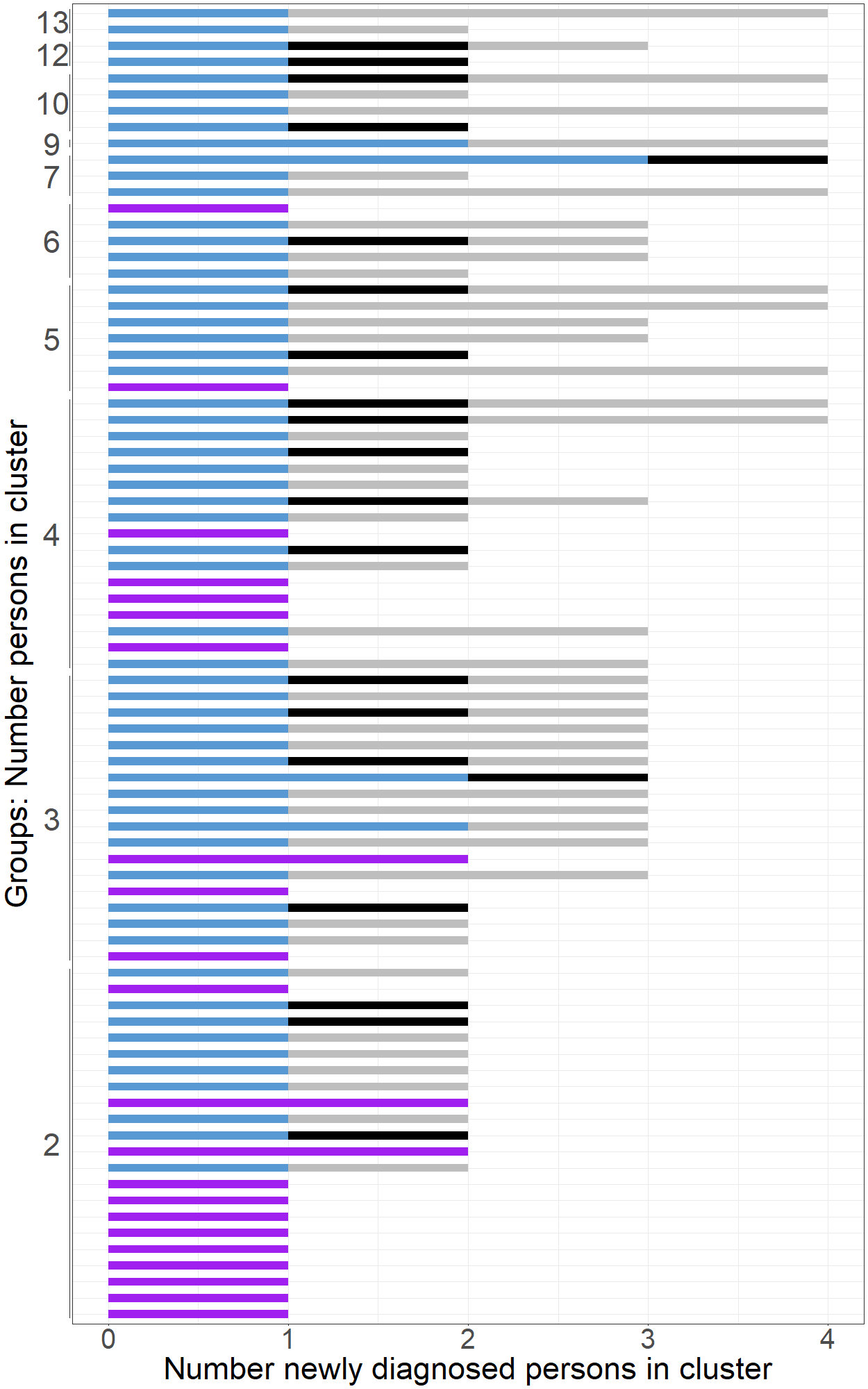

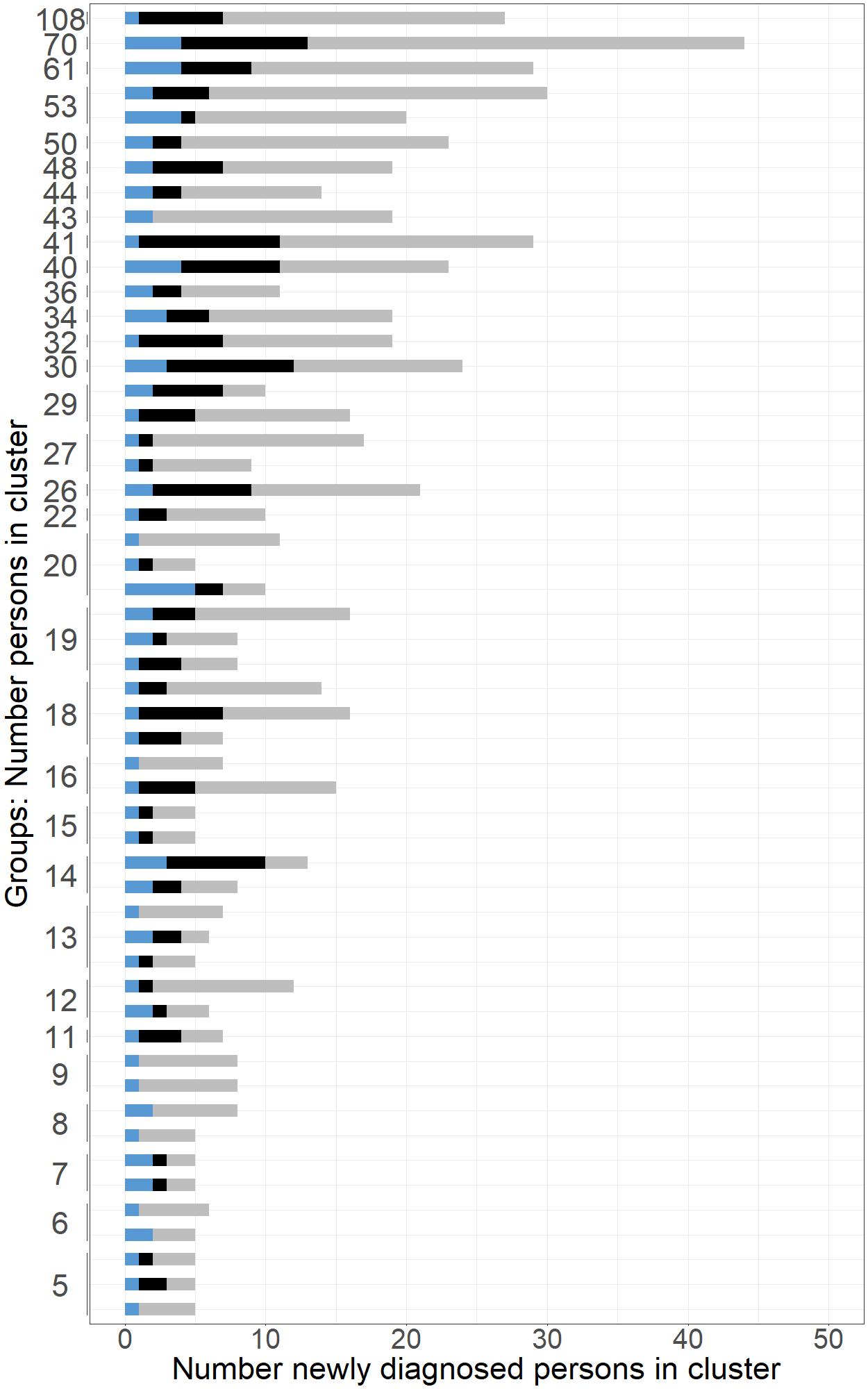
**

**a. Clusters with <5 members newly diagnosed b. Clusters with ≥5 members newly diagnosed**

**Supplemental Figure 2. Characteristics of 134 clusters that included ≥1 member newly diagnosed with HIV by the North Carolina State Laboratory of Public Health (NC-SLPH) with an HIV test sequence but no routine care sequence within one year of HIV diagnosis.** Grey indicates members tested for HIV at a commercial lab, black indicates members tested for HIV by NC-SLPH with an HIV test sequence and routine care sequence, and light blue indicates members tested for HIV by NC-SLPH with an HIV test sequence but no routine care sequence. Highlighted in purple are 22 clusters in which all members newly diagnosed with HIV were tested by NC-SLPH and had an HIV test sequence but no routine care sequence.

**Supplemental Table 1. Eligibility for monitoring of 134 clusters that included ≥1 member newly diagnosed with HIV by the North Carolina State Laboratory of Public Health (NC-SLPH) with an HIV test sequence but no reported routine care sequence.** Active clusters had ≥5 members newly diagnosed between 2018 and 2021, with clusters defined using a genetic distance threshold of <1.5%.

|  | **Non-active cluster:**  **<5 members newly diagnosed**  **(N=81 clusters)** | **Active cluster** | |
| --- | --- | --- | --- |
|  |  | **Already eligible based on available routine care sequences**  **(N=40 clusters)** | **Newly eligible after including HIV test sequences (N=13 clusters)** |
| **Total number members in cluster** |  |  |  |
| Median [Min, Max] | 4 [2, 13] | 24 [6, 108] | 8 [5, 20] |
| **Number members in cluster newly diagnosed** |  |  |  |
| Median [Min, Max] | 2 [1, 4] | 14 [6, 44] | 5 [5, 6] |
| **Number members in cluster newly diagnosed by NC-SLPH with no routine care sequence** |  |  |  |
| Median [Min, Max] | 1 [1, 3] | 2 [1, 5] | 1 [1, 2] |

**Supplemental Table 2. Additional clinical characteristics among people with an HIV test sequence, stratified by whether they had a reported routine care sequence or indicators of HIV care linkage.** HIV test sequences were obtained from remnant serum samples of people testing newly positive for HIV by the North Carolina State Laboratory of Public Health (NC-SLPH) between 2018 and 2021. Follow-up time was within one year of HIV diagnosis for collection of routine care sequence, indicators of linkage to care (defined as a reported HIV viral load or CD4+ T cell count), most recent HIV viral load, and most recent bridge counselor assignment for reengagement.

|  | **Reported routine care sequence** (N=564/847)  **67%** |  | **No reported routine care sequence** (N=283/847)  **33%** | |
| --- | --- | --- | --- | --- |
|  | **Care indicator**  (N = 564/564)  **100%** |  | **Care indicator** (N=220/283)  **78%** | **No care indicator** (N=63/283)  **22%** |
| **Closest viral load to HIV diagnosis date** |  |  |  |  |
| VL <200 | 4 (1%) **^c^** |  | 15 (7%) | 0 (0%) |
| VL 200-9,999 | 82 (15%) |  | 52 (24%) | 0 (0%) |
| VL 10,000-99,000 | 259 (46%) |  | 85 (39%) | 0 (0%) |
| VL >100,000 | 219 (39%) |  | 66 (30%) | 0 (0%) |
| Missing | 0 (0%) |  | 2 (1%) | 63 (100%) |
| **Closest CD4 to HIV diagnosis date** |  |  |  |  |
| CD4 <200 | 81 (14%) |  | 20 (9%) | 0 (0%) |
| CD4 200-499 | 288 (51%) |  | 93 (42%) | 0 (0%) |
| CD4 >=500 | 167 (30%) |  | 96 (44%) | 0 (0%) |
| Missing | 28 (5%) |  | 11 (5%) | 63 (100%) |
| **Patient reports previous positive HIV test?** |  |  |  |  |
| Yes | 56 (10%) |  | 19 (9%) | 8 (13%) |
| No | 459 (81%) |  | 178 (81%) | 37 (59%) |
| Unknown | 3 (1%) |  | 1 (1%) | 1 (2%) |
| Missing | 46 (8%) |  | 22 (10%) | 17 (27%) |
| **Ever assigned bridge counseling for reengagement in care? ^a, b^** |  |  |  |  |
| Yes | 64 (11%) |  | 23 (11%) | 11 (18%) |
| No | 500 (89%) |  | 197 (90%) | 52 (83%) |
| **Bridge counseling for reengagement status** |  |  |  |  |
| Previous diagnosis | 57 (89%) |  | 18 (78%) | 10 (91%) |
| Previous diagnosis, new to NC | 7 (11%) |  | 5 (22%) | 1 (9%) |
| Missing (never assigned) | 500 |  | 197 | 52 |
| **Bridge counseling for reengagement outcome** |  |  |  |  |
| Initiated/renewed | 6 (9%) |  | 1 (4%) | 0 (0%) |
| Refusal | 7 (11%) |  | 1 (4%) | 5 (46%) |
| Unable to locate | 3 (5%) |  | 2 (9%) | 3 (27%) |
| Already in care | 42 (66%) |  | 17 (74%) | 0 (0%) |
| Incarcerated | 1 (2%) |  | 0 (0%) | 1 (9%) |
| Out of state | 1 (2%) |  | 2 (9%) | 1 (9%) |
| Other | 4 (6%) |  | 0 (0%) | 1 (9%) |
| Missing (never assigned) | 500 |  | 197 | 52 |

DEFINITIONS: VL, HIV RNA viral load; CD4, CD4+ T lymphocyte count

**^a^** Most recent bridge counselor assignment for reengagement in care within one year of HIV diagnosis. People were assigned a bridge counselor for care re-engagement if there were no care indicators (HIV clinic visits, CD4+ T cell counts, viral loads, prescription refills) reported to surveillance in the prior 6-12 months.^1^ Disqualifying characteristics for bridge counseling include being already in care, deceased, incarcerated, or not currently living in North Carolina.^1^

^b^ Sample size for ever assigned bridge counseling for reengagement in care is small because outcomes were assessed within one year of HIV diagnosis.

^c^ Percentages may not add to 100 due to rounding

**Supplemental Table 3. Demographic, geographic, and cluster characteristics among people newly diagnosed with HIV between 2018 and 2021 and alive and with an adequate sequence within one year of HIV diagnosis, stratified by HIV test processing location.**

|  | **HIV test processed by a commercial laboratory (N=2,580)** | **HIV test processed by the NC-SLPH (N=847)** | **Total**  **(N=3,427)** |
| --- | --- | --- | --- |
| **Gender ^a^** |  |  |  |
| Man | 2086 (81%) **^g^** | 717 (85%) | 2803 (82%) |
| Women | 457 (18%) | 110 (13%) | 567 (17%) |
| Transgender man | 2 (0.1%) | 1 (0.1%) | 3 (0.1%) |
| Transgender woman | 35 (1%) | 19 (2%) | 54 (2%) |
| **Age at Diagnosis** |  |  |  |
| Median [Min, Max] | 31 [15, 86] | 27 [16, 81] | 30 [15, 86] |
| **Race & ethnicity ^b^** |  |  |  |
| Black, non-Hispanic | 1564 (61%) | 554 (65%) | 2118 (62%) |
| White, non-Hispanic | 575 (22%) | 138 (16%) | 713 (21%) |
| Hispanic | 304 (12%) | 93 (11%) | 397 (12%) |
| Other | 48 (2%) | 17 (2%) | 65 (2%) |
| Missing | 89 (3%) | 45 (5%) | 134 (4%) |
| **HIV risk group ^c^** |  |  |  |
| MSM | 1537 (60%) | 588 (69%) | 2125 (62%) |
| PWID | 77 (3%) | 18 (2%) | 95 (3%) |
| MSM-PWID | 75 (3%) | 22 (3%) | 97 (3%) |
| HET | 606 (24%) | 181 (21%) | 787 (23%) |
| Missing | 285 (11%) | 38 (5%) | 323 (9%) |
| **HIV diagnosis year** |  |  |  |
| 2018 | 546 (21%) | 284 (34%) | 830 (24%) |
| 2019 | 678 (26%) | 243 (29%) | 921 (27%) |
| 2020 | 630 (24%) | 124 (15%) | 754 (22%) |
| 2021 | 726 (28%) | 196 (23%) | 922 (27%) |
| **Diagnosed in North Carolina? ^d^** |  |  |  |
| Yes | 2493 (97%) | 821 (97%) | 3314 (97%) |
| No | 87 (3%) | 26 (3%) | 113 (3%) |
| **Region of residence at diagnosis ^e^** |  |  |  |
| Asheville | 112 (4%) | 61 (7%) | 173 (5%) |
| Charlotte | 772 (30%) | 83 (10%) | 855 (25%) |
| Fayetteville | 244 (10%) | 99 (12%) | 343 (10%) |
| Greensboro | 483 (19%) | 224 (26%) | 707 (21%) |
| Raleigh | 543 (21%) | 191 (23%) | 734 (21%) |
| Wilmington | 128 (5%) | 46 (5%) | 174 (5%) |
| Winterville | 298 (12%) | 143 (17%) | 441 (13%) |

|  | **HIV test processed by a commercial laboratory (N=2,580)** | **HIV test processed by the NC-SLPH (N=847)** | **Total**  **(N=3,427)** |
| --- | --- | --- | --- |
| **Rurality of county at diagnosis ^f^** |  |  |  |
| Rural | 348 (14%) | 171 (20%) | 519 (15%) |
| Urban | 2232 (87%) | 676 (80%) | 2908 (85%) |
| **Identified in a cluster** |  |  |  |
| Yes | 1628 (63%) | 593 (70%) | 2221 (65%) |
| No | 952 (37%) | 254 (30%) | 1206 (35%) |

DEFINITIONS: MSM, men who have sex with men; PWID, person who injects drugs; HET, heterosexual

^a^ Gender was reported as categorical data to NC DPH.

^b^ Race & ethnicity were reported as categorical data to NC DPH and combined into a single variable included in analysis as a proxy for structural racism. We collapsed American Indian/Alaska Native and Asian/Pacific Islander into the Other category due to small cell counts and to match levels used in previous analyses. This group is likely highly heterogeneous.

^c^ HIV risk group was derived by assigning people their most likely transmission category based on exposure data shared during interviews by NC DPH staff at or near the time of diagnosis.

^d^ State of diagnosis could be reported as outside NC if a person was re-tested for HIV at NC-SLPH shortly after relocating to NC or was tested at NC-SLPH but discovered to be residing outside NC.

^e^ Region of residence was based on a person’s county at diagnosis, or upon moving to NC.

^f^ Used the 2013 National Center for Health Statistics Urban-Rural Classification Scheme for Counties to dichotomize county of residence at HIV diagnosis as rural (non-core, micropolitan) or urban (small metro, medium metro, large fringe metro, large central metro).^2^

^g^ Percentages may not add to 100 due to rounding

**REFERENCES**

1. Seña AC, Donovan J, Swygard H, Clymore J, Mobley V, Sullivan K, et al. The North Carolina HIV Bridge Counselor Program: Outcomes From a Statewide Level Intervention to Link and Reengage HIV-Infected Persons in Care in the South. JAIDS J Acquir Immune Defic Syndr. 2017 Sep 1;76(1):e7–14.

2. National Center for Health Statistics. NCHS Urban-Rural Classification Scheme for Counties [Internet]. 2023 [cited 2024 Apr 18]. Available from: https://www.cdc.gov/nchs/data_access/urban_rural.htm
